# Integrative approaches in cardiac tissue engineering: Bridging cellular complexity to create accurate physiological models

**DOI:** 10.1016/j.isci.2025.113003

**Published:** 2025-06-25

**Authors:** Dilip Thomas, Joseph C. Wu

**Affiliations:** 1 Stanford Cardiovascular Institute, Stanford University School of Medicine, Stanford, CA 94305, USA; 2 Division of Cardiovascular Medicine, Department of Medicine, Stanford University School of Medicine, Stanford, CA 94305, USA; 3 Greenstone Biosciences, Palo Alto, CA 94304, USA

**Keywords:** Bioengineering, Biomaterials, Cardiovascular medicine, Tissue engineering

## Abstract

Recent innovations in cardiac tissue engineering (TE) have yet to fully harness integrative genomic mapping of cellular niches to replicate the spatially organized cellular communities and extracellular matrix (ECM) microniches of the heart. Bridging this gap will allow the development of robust platforms for cardiac regeneration and disease modeling. Recapitulating this complexity, including hierarchical vascularization, functional innervation, and immune integration, remains a fundamental challenge in precision cardiac tissue engineering. While iPSC-derived models, engineered biomaterials, and multi-scale 3D bioprinting have advanced creation of cardiac constructs, most of them still lack the optimal maturity and functional multicellular crosstalk. To address these gaps, this review critically evaluates our current understanding of cardiac cellular/ECM heterogeneity and synthesizes progress in recapitulating these features. By aligning challenges with emerging innovations, we provide a roadmap to drive cardiac tissue engineering innovations toward clinically transformative solutions.

## Graphical abstract

**Figure:**
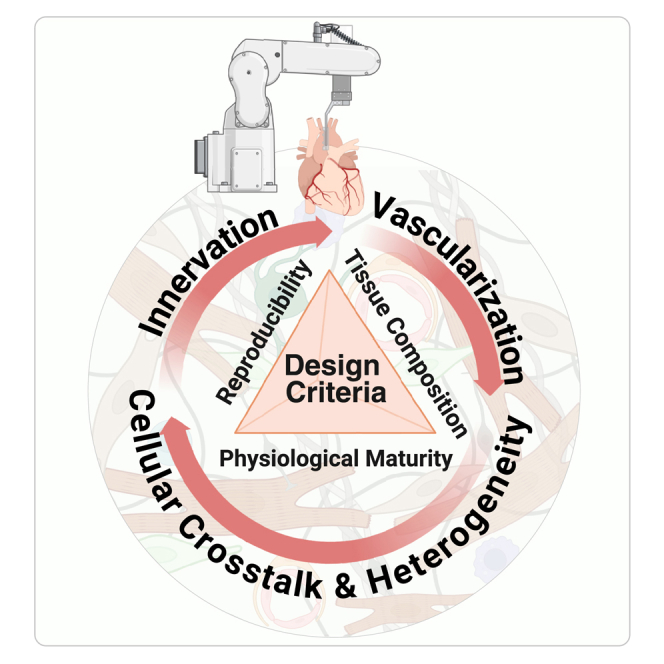

## Introduction

Recent advancements in population-wide integrative genomics and spatial mapping technologies have propelled our understanding of cellular dynamics in cardiovascular health and disease. These technologies offer novel insights into cellular diversity and its intricate microenvironments within the cardiac tissue.^1^ Despite advancements, translating these findings into clinical applications remains limited by the inherent shortcomings of current preclinical models. Clinical success relies on precise mechanistic understanding derived from model systems that accurately mirror cell identities, function, and pathophysiological mechanisms.^2^^,^^3^ Conventional approaches for studying key mechanisms are often limited to specific cell types, microenvironments, and perturbations. Recent spatial mapping techniques studies have contributed to our understanding of the complexities of the human heart, which is characterized by diverse cellular and extracellular components that work in concert to sustain its mechanical, electrical, and metabolic demands.^4^^,^^5^^,^^6^ Hence it is essential to study multiple aspects of the disease that are influenced by cellular heterogeneity and dynamic changes in the tissue microenvironment. Harnessing the power of humanized model systems and cross-species pre-clinical model systems can better address the differences arising due to cell types, pathophysiological responses, drug interactions, and temporal shifts in tissue niches. Novel bioengineering approaches offer promise in predicting structure-function relationships by combining biological insights, advanced materials, and scalable technologies through fabrication of functional cardiac constructs for regenerative medicine, disease modeling, and drug discovery.^7^^,^^8^^,^^9^

**Figure 1:**
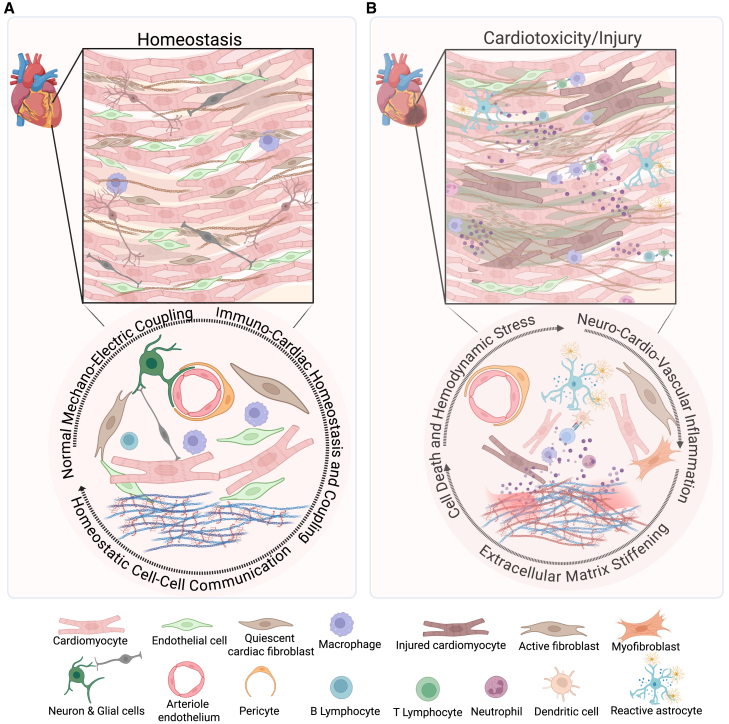
Dynamic cellular landscape of cardiac tissue microenvironment in states of homeostasis and injury (A) Various cell types in the heart, including cardiomyocytes, endothelial cells, fibroblasts, neurons, and tissue-resident macrophages, engage in active crosstalk to maintain a homeostatic myocardial niche and extracellular matrix integrity. (B) Following injury, interstitial fibroblasts enter a sustained activation state, accompanied by immune infiltration, cell death, and localized neurovascular inflammation. Cardiac injury signatures vary across cardiovascular pathologies, exhibiting distinct patterns of cellular and extracellular remodeling. Recapitulating the biochemical and biophysical interactions between cells and the extracellular matrix is essential for modeling native and pathological states of the heart.

This review explores the strategic integration of scalable technologies in cardiac tissue engineering, with a focus on replicating cellular heterogeneity, developing hierarchical vascularization, and incorporating functional innervation and immune components. Cardiomyocytes, the contractile cells of the myocardium, are embedded within a matrix of endothelial cells, fibroblasts, and immune cells, each contributing to the organ’s dynamic functionality. Single-cell transcriptomics and spatial omics are being used to investigate the heterogeneity and spatial organization of these cellular populations, revealing their specialized roles and interactions within distinct anatomical regions at scale.^1^^,^^10^ Similarly, the extracellular matrix (ECM) plays a critical role in providing structural support, mediating cell signaling, and maintaining tissue homeostasis.^11^^,^^12^^,^^13^ Understanding these cellular and ECM interactions is essential for designing engineered tissues that accurately replicate the physiological microenvironment of the heart (Figure 1). Under physiological homeostasis, complex interactions among cell types, including cardiomyocytes, endothelial cells, fibroblasts, and glial cells to modulate the heart’s mechanical and electrical function, whereas immune cells offer immunosurveillance to clear cellular debris and resolve inflammatory responses (Figure 1A). Cardiac stress and injury lead to altered cell-cell interactions driven by activation and recruitment of immune cells, disruption in electrical coupling, and excessive deposition of ECM by fibroblasts (Figure 1B).

Over the past decade, the convergence of stem cell biology and bioengineering has enabled the development of sophisticated cardiac models. Induced pluripotent stem cells (iPSCs) have revolutionized cardiovascular research by providing a renewable, patient-specific source of cardiovascular cell types.^7^^,^^14^^,^^15^^,^^16^ Advances in differentiation protocols now enable the generation of chamber-specific subtypes, closely mimicking the anatomical and cellular identities of the native heart.^7^ However, inducing these cells into functional tissue requires overcoming challenges in structural organization and maturation. To address this gap, bioengineering technologies such as advanced three-dimensional (3D) scaffold fabrication and tailored maturation strategies offer transformative potential for achieving functional integration and regeneration of cardiac tissue.^17^^,^^18^ Techniques that rely on temporal delivery of morphogenetic cues to cardiac progenitors within spatial confinement can help create self-assembled cardiac constructs, providing insights into cellular interactions and gene regulation during development or disease states.^7^^,^^8^^,^^19^ A major challenge in cardiac tissue engineering is the recapitulation of functional tissue vascularization and innervation.^20^ Matching the high metabolic demands of the heart tissue require a dense and hierarchical vascular network capable of delivering oxygen and nutrients, while simultaneously maintaining a time-resolved equilibrium in structural integrity. Advanced bioprinting and sacrificial templating techniques have made significant strides in fabricating perfusable microvascular networks, but achieving scalability and physiological fidelity remains critical challenges. Beyond vascularization, the incorporation of innervation and immune components is essential for creating truly functional cardiac tissues.^21^ Neural integration modulates cardiac excitability and rhythm, whereas immune cells play a dual role in tissue repair and inflammation, highlighting the importance of these systems in both healthy and disease states. The clinical and industrial-scale translation of cardiac tissue engineering depends on developing scalable fabrication processes capable of achieving physiological myocardial maturation, as well as integrating multimodal systems like vasculature, nerves, and immune components.

By reviewing recent advancements in stem cell biology, biomaterials, and biofabrication techniques, we aim to highlight the transformative potential of engineered cardiac tissues in advancing both regenerative medicine and cardiovascular research. We address current challenges and identify future directions to underscore the critical role of multidisciplinary collaboration in realizing the promise of cardiac tissue engineering.

## Understanding cellular and extracellular composition of the heart: A foundation for cardiac tissue engineering

The heterogeneity in cellular composition in the human heart has been widely contested in the field for decades. Using stereological and flow cytometry-based assays, previous studies estimated the proportions of major cell types of the heart, with ∼20%–30% being cardiomyocytes, ∼25% being endothelial cells, and ∼40%–50% being mesenchymal cell populations.^22^^,^^23^ More advanced characterization tools and genetically encoded probes that can identify cell-specific protein expression now allow the cellular composition question to be revisited using murine model and adult human heart slices. The study found 32% cardiomyocytes, 55% endothelial cells (ECs), and 13% cardiac fibroblasts (FBs), which was consistent with previous studies, except for CD45^+^ leukocytes that were reported to be 3 times more in the murine heart.^24^

More recently, detailed studies using single-cell or single-nucleus transcriptomics have created comprehensive human heart atlases^4^^,^^5^^,^^6^^,^^25^ mapping six distinct anatomical domains including: the left ventricle, right ventricle, right and left atria, apex and interventricular septum. These techniques provide higher resolution in defining the composition within the defined anatomical regions. For example, one of the earlier studies highlights the differences in distinct cell proportions between atrial and ventricular tissues. Atrial tissues contain approximately 30% atrial cardiomyocytes, 12% endothelial cells, 24% fibroblasts, 17% mural cells, and 10% immune cells from myeloid and lymphoid origin, whereas ventricular tissues comprise of approximately 49% ventricular cardiomyocytes, 8% endothelial cells, 15% fibroblasts, and 5.3% immune cells.^4^ In a developing human heart, the diversity in cardiomyocytes is highly stratified based on the morphological structure and the regions where they reside. Single-cell RNA sequencing (scRNA-seq) and high-resolution mapping of developing human hearts were used to generate a map with a finer resolution to distinguish distinct cell populations based on distinct anatomical domains of the heart undergoing multicellular interactions to become organized cardiac structures.^6^

Although it is unclear what drives spatiotemporal signaling, it is evident that the specialization of every functional cellular ecosystem is driven by its own highly complex genetic program, which is then translated into multicellular signaling pathways to allow tissue-level organization and morphogenesis.^26^^,^^27^ For example, the spatially localized expression of transcriptional regulators *NR2F1*^+^ and *IRX4*^+^ drives formation of atrial and ventricular chambers, respectively. During development, atrial myocyte population was found to be less diverse with only two structurally distinct populations distinguished by *ANGPT1* expression, whereas ventricular myocytes were identified based on their fate in becoming either compact or trabecular myocytes with *HEY2*^*+*^ or *IRX3*^*+*^ expression, respectively. The hierarchical compartmentalization of these cells occurs mostly in the left ventricle during development followed by right ventricle through transitional appearance of compact and trabecular myocyte hybrids. By contrast, previously uncharacterized populations of non-chambered myocytes (*BMP*^*+*^*/CNN1*^*+*^*/CRABP2*^*+*^) and heterogeneous non-myocyte cellular populations drive the formation of inflow/outflow tract, pacemaker regions, and heart valves.^6^

The heart chambers are intricately connected by the heart rhythm headquarters housed within the cardiac conduction system comprising of sinoatrial (SA) and atrioventricular (AV) nodes. Pacemaker cells within the heart are identified by the expression of *ISL1*, *SHOX2*, *TBX3*, and *TBX18*. Recent studies highlighted the importance of a well-conserved Ca^2+^ binding protein Visinin-like 1 protein (*VSNL1*) as a specific marker for the SA node across various mammalian systems.^28^^,^^29^ During early development of SA node, there is active suppression of working myocardium (downregulating *Nkx2-5*) with simultaneous activation of ion channel genes.^30^ Advances in spatial transcriptomics now allow mapping of fine-grained cell states within the sinoatrial-atrioventricular (SA-AV) region. This technique has identified cell populations expressing classical pacemaker genes (e.g., *HCN1* and *HCN4*) and ion channels essential for cardiac rhythm regulation.^31^ The AV bundle cells, identified by the enrichment of *GJA5*, *CRNDE*, and *CNTN5* markers, share similarities with the pacemaker cells in their expression of ion channels. However, unlike the SA node’s low conductance connexin (Cx45), these cells express high conductance connexins (Cx40 and Cx43). This distinction enables the AV bundle to propagate electrical impulses to the peripheral Purkinje fibers, a network of specialized myocytes extending into the ventricular apex. These fibers are critical for synchronizing ventricular contraction to express signature gene markers (*IRX3*, *KCNJ3*, and *MYL4*) that regulate their unique electrophysiological properties.^31^

While the coordinated activity of pacemaker cells, Purkinje fibers, and connexins ensures precise electrical signaling, the immune cells play a critical role in responding to injury and maintaining tissue homeostasis. Although sparse in a healthy tissue, immune cells have dynamic roles in cardiovascular remodeling.^4^ Recent single cell studies identified 13 distinct populations of myeloid cells and 8 cell clusters of lymphoid origin. Most of these immune cells likely arise from the circulation except *LYVE1*^+^ (lymphatic vessel endothelial hyaluronan receptor 1) expressing tissue resident macrophages (MΦs) and *LYVE1*^−^ antigen presenting MΦs. Heterogeneity in the macrophage populations is due to differentiation from monocytic pre-cursors under specific immunogenic stimulus or disrupted homeostatic interactions with myocytes or non-myocytes.

All cardiac cells, including immune populations, are enmeshed within an ECM that provides anchorage, guidance, and insulation against hyperpolarization due to electrical overload. This ECM is particularly vital in the healthy adult myocardium, where cardiomyocytes are embedded in a pliable matrix that balances mechanical compliance with structural resilience. The myocytes largely anchor to collagen fibers that are synthesized by FBs, with collagen types I and III comprising over 90% of the total composition.^12^ In addition to collagens, other key matrix components include fibrillins, laminins, and proteoglycans necessary for structural support in both cardiomyocytes and nonmyocytes.^32^ These ECM components are organized mainly in the pericellular and interstitial regions, and the intertwined pericellular matrix helps maintain cell polarity and establish direct integrin-mediated anchoring via integrins and surface receptors. The interstitial matrix provides a foundation for intercellular junctions for electrical coupling and a channel for the transportation of molecules that mediate cellular responses in normal as well as diseased states.^33^

The plasticity and conductivity of the ECM is attributed to the glycosylation of the structural proteins. Proteoglycans, glycoproteins, and glycosaminoglycans in the basement membrane play an important role in maintaining homeostasis and cardiac remodeling during development and injury repair. Structural, fibrillar ECM components serve as a scaffolding barrier, whereas non-fibrillar charged proteoglycans act as an electrostatic “sieve” to determine the trafficking of solutes through the matrix.^34^ Glycans that are bound to these proteins serve various functions by providing protein stability, sequestration of growth factors, and cytokines for signal transduction.^35^ For example, heparin sulfate proteoglycans (HSPGs), such as syndecan, hyalectans, and glypicans are important for development and disease. The glycoproteins mainly act via shedding or binding to the ECM, which elicits a response by targeting immunomodulatory cells. For example, the binding of versican, a hyaluronan binding proteoglycan, promotes leukocyte adhesion in myocarditis,^36^ whereas the presence of soluble syndecan-4 is correlated with an inflammatory response in the failing heart.^37^ Deletion or mutation in HSPGs can modulate the binding affinity and release kinetics of growth factors, thus influencing their bioavailability during key compensatory physiological processes in tissue remodeling.^38^

In addition to cytokine and chemokine-triggered signaling responses, ECM proteolysis via transmembrane interactions plays an important role in driving signaling responses. One of the most studied transmembrane glycoproteins, extracellular matrix metalloproteinase inducer (EMMPRIN), can mobilize monocytes and leukocytes to induce their differentiation into MΦs. Interactions of EMMPRIN with several glycoproteins and ligands result in specific biological function under physiological and pathological remodeling. For example, translocation of EMMPRIN to the cell surface by the plasma membrane protein caveolin-1 stimulates proteolytic cleavage. Depending on the degree of EMMPRIN glycosylation, higher glycosylation induces stronger proteolysis compared to low or deglycosylated forms. During ischemia or tissue injury, there is an increase in expression of highly glycosylated EMMPRIN that leads to a proinflammatory cascade. In contrast, EMMPRIN interaction with cyclophilin B can trigger angiogenesis and confer protection against oxidative stress through activation of the Erk pathway.^33^ Similar to EMMPRIN, thrombospondins are another class of matricellular proteins that bind to major structural proteins and integrins. Particularly in the heart, matricellular proteins, such as thrombospondins, periostin, and tenascins can induce matrix deposition via TGF-β1 (transforming growth factor β1) activity and antagonize matrix degradation by inhibiting a broad range of proteases.^11^ Due to their tightly bound associations with the ECM, these components exert an adaptive mechanosensitive function under conditions of acute myocardial stress. Given these critical roles, the native ECM ultrastructure and composition provide the foundation for bi-directional mechano-signaling and help with dynamic remodeling during development and disease. This intricate role of the natural ECM in cardiac function has inspired efforts to replicate its properties in engineered biomaterials, aiming to create functional cardiac tissues.

Considering the complexity of the heart and its ever-changing cellularity and ECM landscape, it is essential to engineer the most representative myocardium with seamless integration of cardiomyocytes and other supportive cell types to generate functional heart tissue for testing effective therapies and long-term strategies, with the goal of augmenting heart function with viable *ex vivo* generated cardiac tissue grafts. The past several decades have seen the precise deciphering of signaling pathways and tracing of cellular trajectories that led to the development of refined protocols to generate most cardiac cell types from iPSCs. Studies that now exclusively use human myocytes and non-myocytes to facilitate cell-cell communication and interaction in an engineered tissue microenvironment are summarized in Table 1. The use of these cell types in a top-down manner has enabled various tissue fabrication technologies in the field of tissue engineering, including the development of cardiac tissue-like architecture and incorporation of pre-differentiated heart cells to promote formation of heart tissue. iPSCs can be differentiated into all major cell types in the heart with varying degrees of purity and maturity. The development of cardiovascular cell types from iPSCs involves sequential formation of mesoderm, cardiac mesoderm, and cardiac progenitors, as well as the activation of evolutionarily conserved cell-specific transcription factors. Detailed information on the latest developments in generating chamber-specific myocytes and non-myocytes is comprehensively reviewed elsewhere.^7^^,^^8^

**Table 1:** Summary of the most recent biofabrication technologies for generating human tissue-engineered cardiac constructs with defined cellular and matrix compositions

Platform	Technique	Structure/Cell numbers			Tissue composition	Support matrix	Reference
		Format	Size	Cell numbers			
**Cell Self-assembled Cardiac Models**							

Microtissues	U-bottom microplate aggregation	Spheroid	∼250–300 μm	1 × 10^3^/spheroid	hESC-CMs, fetal CFs, hCMECs (4:2:1)	–	Ravenscroft et al.^39^
Microtissues	V-bottom microplate aggregation	Spheroid	∼250–300 μm	5 × 10^3^/spheroid	hiPSC-CMs, hiPSC-ECs (85%:15%)	–	Giacomelli et al.^40^
Microtissues	U-bottom agarose mold aggregation	Spheroid	∼250 μm	∼1.5 × 10^5^/spheroid	50% hiPSC-CMs and 50% non-myocytes (4:2:1 ratio of hCFs, HUVECs, hADSCs)	–	Richards et al.^41^
Microtissues	U-bottom agarose mold aggregation	Spheroid	∼150 μm	∼1.5 × 10^5^/spheroid	50% hiPSC-CMs and 50% non-myocytes (4:2:1 ratio of hCFs, HUVECs, hADSCs)	–	Richards et al.^42^
Microtissues	Hanging drop microplate aggregation	Spheroid	∼400 μm	1 × 10^4^/spheroid	hiPSC-CMs, ECs, hiPSC-CFs (2:1:1)	–	Polonchuk et al.^43^
Microtissues	U-bottom microplate aggregation	Spheroid	∼200 μm	0.5 × 10^3^/spheroid	hiPSC-CMs, CF, hCMEC (4:2:1)	–	Pointon et al.^44^
Microtissues	U-bottom microplate aggregation	Spheroid	∼250 μm	1 × 10^3^/spheroid	hiPSC-CMs, hCFs (90%:10%)	–	Devarasetty et al.^45^
Microtissues	V-bottom microplate aggregation	Spheroid	∼400 μm	5 × 10^3^/spheroid	hiPSC-CMs, hiPSC-EC, hiPSC-hCFs (70%:15%:15%)	–	Giacomelli et al.^46^
Microtissues	U-bottom agarose mold aggregation	Spheroid	300–400 μm	∼1 × 10^6^ cells mL^−1^	hiPSC-CMs, hiPSC-ECs (4:1)	–	Giacomelli et al.^47^
Microtissues	U-bottom microplate aggregation	Spheroid	150–200 μm	∼1.2 × 10^4^ cells mL^−1^	hiPSC-CMs, hCMECs, and hCFs (4:2:1)	–	Archer et al.^48^
Microtissues	U-bottom agarose mold aggregation	Spheroid	300–400 μm	∼5.5–11 × 10^3^ cells mL^−1^	hiPSC-CMs, hCFs (95%:5%)	–	Campostrini et al.^49^
Microtissues	Bioprinting	Spheroid Patch	∼500 μm/spheroid ∼3 mm × 3 mm	3.3 × 10^5^/spheroid	hiPSC-CMs, hCFs, HUVECs (70%:15%:15%)	–	Ong et al.^50^
Microtissues	Needle Array Bioprinting	Spheroid, Frame patch	∼1 mm × 1 mm	3.5 × 10^4^/spheroid	hiPSC-CMs, HUVECs, NHDFs (50%:25%:25%)	–	Arai et al.^51^

**ECM-based Cardiac Models**							

EHT	PDMS Pillar Suspension	Strip	∼6 × 1.8 mm	2 × 10^6^ cells/tissue	hiPSC-CMs, HDFs (75%:25%)	Fibrin	Ronaldson-Bouchard et al.^52^
EHT	Heart Dyno Insert	Strip	∼1.3 × 0.5 mm	5 × 10^4^ cells/tissue	hiPSC-CMs, Stromal cells (70%:30%)	Collagen I, Matrigel	Mills et al.^53^
EHT	Biowire	Strip	5 mm × 1 mm × 300 μm	1 × 10^5^ cells/tissue	hESC-CMs/hiPSC-CMs, hCFs (75%:25%)	Collagen/Matrigel/Fibrin	Zhao et al.^54^
EHT	PDMSInsert	Strip	∼3 × 5–9 mm	1.6 × 10^6^ cells/tissue	hESC-CMs/hiPSC-CMs, hCFs (75%:25%)	Collagen I, Matrigel	Abilez et al.^55^
EHT	PDMS Pillar	Strip	∼0.5 × 0.5 × 6 mm	1.5 × 10^6^ cells/tissue	hESC-CMs/hiPSC-CMs, skin FBs, gingiva- and hCFs (70%:30%)	Collagen I, Matrigel	Tiburcy et al.^56^
EHT	PDMSInsert	Ring	∼0.5 × 0.5 × 6 mm	2 × 10^6^ cells/tissue	hESC-CMs (atrial and ventricular)	Collagen I	Goldfracht et al.^57^
EHT	Heart Dyno Insert	Strip	∼1.3 × 0.5 mm	∼5 × 10^4^ cells/tissue	hiPSC-CMs, hiPSC-ECs (80%:20%)	Collagen I, Matrigel	Mills et al.^58^
EHT	PDMS insert	Ring	∼1 cm diameter	∼1.5 × 10^5^ cells/tissue	hiPSC-CMs, NHDF (3:1)	Polyethylene glycol	Seguret et al.^59^
μEHT	PDMS insert/cell aggregation	Dogbone	∼0.1 mm shaft, 0.5 mm knob	∼2 × 10^7^ cells mL^-1^	hiPSC-CMs, EB-CFs (1:1)	–	Huebsch et al.^60^
EHT	PDMS insert	Strip	Three sizes: 130 × 485 × 139 μm, 1410 × 629 × 185 μm, 1548 × 657 × 192 μm	∼1.57 × 10^7^ cells mL^−1^	hiPSC-CMs, hiPSC-ECs, hiPSC-CFs (70%:15%:15%)	Collagen I, Matrigel	Windt et al.^61^
μEHT	PDMS insert	Strip	∼3 mm × 0.1 mm	∼59 × 10^3^ cells/tissue	hiPSC-CMs, hiPSC-ECs, hiPSC-SMCs hUCFs (70:19:4:7)	Fibrin, Matrigel	Cofiño-Fabres et al.^62^
Microtissues	Extrusion printing	Cylindrical and Sheet	∼14 mm × 4 mm	1 × 10^6^ cells mL^−1^	iPSC-CMs, hNDFs (9:1)	Gelatin, Fibrin, Gelbrin (gelatin-fibrin)	Ahrens et al.^63^
Microtissues	Extrusion printing	Frame patch	∼1 × 1 mm (∼200–250 μm/spheroid)	1 × 10^7^ cells mL^−1^	hESC-CMs and hESC-ECs (7:3)	Fibrin, Gelatin, Hyaluronic acid	Liu et al.^64^

**Tissue Chip Cardiac Models**							

Microfluidic tissues	Flow mediated cell seeding/self-assembly	Multi- cell layers	–	1 × 10^8^ cells mL^−1^	hiPSC-CMs, HUVECs, hCFs (1:1:0.1)	Fibrin	King et al.^65^
Microfluidic tissues	Flow mediated cell seeding/self-assembly	Dogbone	∼1 mm shaft, ∼0.6 mm knob	4 × 10^7^ cells mL^−1^	hiPSC-CMs, hCFs (1:10), hiPSC-ECs lined (5 × 10^6^ cells mL^−1^)	Fibrin, Collagen I, Matrigel	Vivas et al.^66^
Microfluidic tissues (InVADE/AngioTube)	Chamber/Flow mediated cell seeding/self-assembly	Strip	∼1 mm × 0.6 mm	1.5–2 × 10^6^ cells/tissue	iPSC-CMs (100 × 10^6^ cells mL^−1^, HUVECs lined (25 × 10^6^ cells mL^−1^) (4:1)	Fibrin, Gelatin	Lai et al.^67^
Microfluidic tissues	Flow mediated cell seeding	Multi- cell layers	–	7 × 10^6^ – 2 × 10^7^ cells/tissue	hiPSC-CMs (1.4–4× 10^7^ cells mL^−1^), hiPSC-ECs lined (3 × 10^7^ cells mL^−1^)	Gelatin methacryloyl	Ellis et al.^68^
Microfluidic tissues	Confined cell seeding	Multi- cell layers	–	35 × 10^6^ cells mL^−1^	hESCs/hiPSC-CMs, hCFs (4:1)	Collagen, Matrigel	Veldhuizen et al.^69^

hESC, human embryonic stem cell; hiPSC, human induced pluripotent stem cell; EB, embryoid body; ECM, extracellular matrix; EHT, engineered heart tissue; CM, cardiomyocyte; EC, endothelial cell; FB, fibroblast; SMC, smooth muscle cell; hADSC, human adipose derived stromal cell; HDF, human dermal fibroblast; hCFs, human ventricular fibroblast; hUCF, human uterine fibroblast; hCMEC, human microvascular endothelial cell; HUVEC, human umbilical vein endothelial cell.

## Recapitulating cellular diversity for *in vitro* and *in vivo* engineered cardiac tissues

Recapitulation of biological complexity of the heart using tissue engineering approaches has evolved in parallel based on the context of use, ranging from *in vitro* miniature tissues for drug testing and disease modeling to the generation of myocardial patches for repair and restore cardiac function *in vivo*, and more recently, organized efforts in creating organ-scale cardiac tissue enabled by advancements in bioprinting technologies. These diverse applications are based on different design strategies that prioritize cellular fidelity, reproducibility, or the ability to recapitulate pathological phenotypes at a smaller scale for *in vitro* disease modeling. In contrast, *in vivo* the primary challenge lies in achieving seamless integration with the host tissue, requiring constructs that not only mimic the mechanical and electrophysiological properties of native myocardium, but also actively promote vascularization and electromechanical coupling.

**Figure 2:**
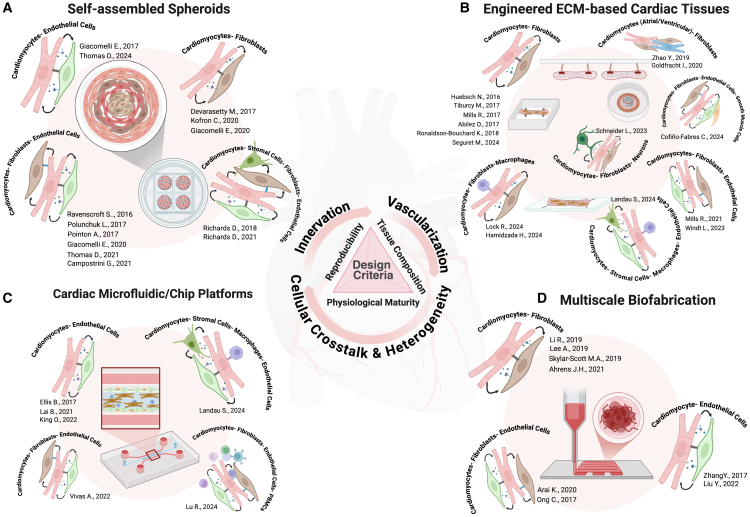
Mimicking cellular heterogeneity in tissue-engineered cardiac constructs facilitates the creation of cardiac niches that promote cell-cell communication (A–D) This schematic summarizes key platforms and technologies that support static and dynamic cell-cell interactions in (A) microtissues, (B) engineered heart tissues, (C) microphysiological systems, and (D) bioprinted platforms. Employing facile biofabrication design strategies to integrate diverse cardiovascular cell types with functional innervation and vascularization is crucial for unraveling cell-cell interaction-mediated transitions during disease onset and in response to therapeutic interventions in disease modeling.

Tissue engineering technologies tailored to the field can be broadly classified into two main categories: (1) top-down models that employ tissue-like scaffolds with integrated stimulation components or sensors to capture cardinal physiological responses; and (2) bottom-up models that rely on forced self-assembly or morphogenetic factors driving cell-guided assembly to form primitive, less sophisticated cardiac architecture. Top-down models in tissue engineering aim to mimic several attributes of cardiac tissue to enhance maturity and function by tuning mechanical properties, tissue architecture, direction of mechanical load, and electrical stimulation. By comparison, bottom-up approaches aim to mimic the cellular and extracellular heterogeneity for optimal cell-cell interaction or dysfunction by simulating physiological or pathological reactions in response to a stimulus or genetic mutation. Emerging *in vitro* systems, such as self-assembled spheroids, microphysiological systems (MPS), ECM-supported cardiac tissues, and 3D bioprinting aim to mirror the human heart’s biological complexity (Figure 2). These systems, when designed with precision, coordinate a symphony of cell-cell communication, vascularization, and controlled maturation, bridging the gap between simplicity and clinical relevance. Self-assembled cardiac spheroids (Figure 2A) leverage cell-cell interactions to form 3D aggregates that better replicate compact cell-cell adhesion than monolayer cultures. These spheroids often combine multiple major cell types, such as CMs, ECs, and FBs to simulate paracrine signaling and mechanical coupling observed *in vivo.* Unlike developmental organoids, which rely on stochastic self-organization, spheroids enable precise control over cell composition, mitigating heterogeneity in differentiation outcomes.^19^ A key limitation of conventional organoids is their metabolic immaturity. Immature iPSC-CMs predominantly rely on glycolysis due to persistent HIF1α-LDHA axis activation, mirroring fetal rather than adult metabolism.^70^ To address this, recent protocols employ metabolic priming strategies, such as glucose-free media supplemented with fatty acids (palmitate, oleate) and maturation-promoting metabolites (creatine, L-carnitine, taurine).^71^^,^^72^ Alternatively, pharmacological inhibition of HIF1α-LDHA has shown to further accelerate maturation, yielding CMs with hyperpolarized resting membrane potentials, faster upstroke velocities, and increased force generation.^70^

In addition to these chemical and electromechanical techniques, the incorporation of non-myocytes has been shown to play indispensable roles in cardiac maturation in 3D, both structurally and functionally.^16^ For example, FBs have been shown to enhance CM function through elevation of cardiac contractile proteins, and paracrine signaling via secretion of VEGF-mediated Cx43 expression in CMs, improving electrical coupling and conduction velocity.^73^^,^^74^ Additionally, in FB-EC-CM co-culture, it is proposed that FB-derived cGMP may enhance cAMP-mediated maturation signals in CMs, whereas EC-derived endothelin-1 (EDN1) may activate adenylyl cyclase, further amplifying cAMP-dependent pathways.^46^ ECM-based cardiac tissues are inspired by nature’s blueprint, leveraging natural or synthetic matrices to guide cell behavior (Figure 2B). For example, the use of collagen as an ECM support replicates the heart’s anisotropic stiffness, directing cells to organize into electromechanically coupled tissues that conduct electrical impulses with near-physiological efficiency.^56^ These constructs typically are anchored to deformable substrates to further allow fine-tuning of stretch-dependent preload and afterload conditions.^75^ Such dynamic mechanical tuning not only enhances maturation but also models pathological remodeling, such as fibrosis or diastolic dysfunction.

MPS, or “heart-on-a-chip” platforms, combine microfabrication and tissue engineering to simulate mechanical and biochemical cues with structural features embedded within the chips (Figure 2C). These microfabricated devices subject cardiac tissues to rhythmic stretching and fluid flow, simulating the mechanical stresses of a beating heart. Perfusable channels lined with ECs mimic blood circulation^76^ to deliver nutrients and shear stress cues, and are useful for immune cell recruitment studies.^77^ Among tissue engineering strategies, 3D bioprinting is the most scalable approach, enabling precise layer-by-layer deposition of cells and bioinks positioned like living pixels (Figure 2D). This technique allows easy embedding of vascular channels by virtue of sacrificial templating and assembly of ECs in spatially defined zones.^78^ While bioprinted cardiac tissues at millimeter scales demonstrate synchronized beating and action potential propagation, scaling to human-sized hearts demands greater leaps in biofabrication capability (discussed further in Section from building blocks to the development of 3D chamber-like architecture using tissue engineering techniques).

In recent years, the generation of tailored, small-scale cardiac patches using human iPSC-CMs to remuscularize the heart after myocardial infarction or heart failure has shown significant promise as a therapy. The evidence suggests cardiac patches seeded with multiple cell types have higher regenerative potential. For example, a fibrin-based patch seeded with iPSC-CMs, ECs, and SMCs were shown to achieve engraftment with higher vascularization and improvement ejection fraction in a porcine ischemia reperfusion mode.^79^ Similarly, in a cardiac cryo-injury guinea pig model, fibrin patches laden with iPSC-CMs and ECs improved re-muscularization of the infarct area and ejection fraction by 31%.^80^ Similarly, a mixed culture of iPSC-CMs, SMCs, and FBs on a fibrin-based scaffold fabricated using a molding technique was shown to improve maturation of the myocytes with contractile forces of ∼5 mN and conduction velocity of ∼26 cm/s. These matured patches when tested for 3 weeks in an *ex vivo* rat heart model showed successful engraftment without abnormal conduction defects in the host tissue due to insufficient electrical coupling.^81^ Despite high relevance and applicability, the efficacy of these engineered constructs need to be demonstrated next in large animal models, such as pigs or non-human primates. Besides the infarction-induced hostile tissue environment, the two major challenges in large-animal model transplantation are the requirement for frequent immunosuppression regimens to allow engraftment, and matching graft strength to the stronger contractile forces.

Querdel et al. performed a dose-dependent study with the implantation of fibrin-based scaffold with immunosuppression in cryoinjury guinea pig compared to a healthy pig model. The patches composed of human iPSC-CMs at dose of ∼9-15 × 10^6^ for the guinea pigs and 450 × 10^6^ cells for the pig model. The highest cell dose was shown to be efficacious in improving the ejection fraction by 8% compared to post-injury values with the guinea pigs. In the pig model, the grafts withstood the mechanical forces and showed cardiomyocytes persistence after 4 weeks.^82^ For clinical applications, the use of fibrin to generate patches may be limiting as it is a blood-derived component and may further exacerbate immune-mediated adverse responses. Instead, the use of collagen as a scaffolding matrix that is abundant in myocardium may impart superior mechanical strength and physiological tissue microenvironment. In a recent long-term dose-dependent study, Jebran et al. demonstrated efficacy in a rhesus macaque myocardial infarction model in which the animals were implanted with a medical grade collagen-based patch constructed from 2.64 × 10^9^ human iPSC-CMs and stromal cells. The macaques showed enhancement in wall contractility and ∼7% relative improvement in ejection fraction. Although some vascularization of the graft was observed, it was lower than the surrounding healthy muscle. Finally, the authors were able to apply an allograft cardiac patch (8 × 10^8^ cells) on to a human heart of a patient enrolled in Bio-VAT-HF clinical trial. Bio-VAT-HF (NCT04396899) is an ongoing first-in-human cardiac patch trial for heart failure patients. After the patient obtained a healthy transplant at three months, the patch-engrafted failing heart was explanted for analysis. Similar to the macaque hearts, the patch CMs were smaller and immature compared to host cells and exhibited reduced vascularization and mild infiltration of immune cells. Other proof-of-concept *in vitro* studies^46^^,^^73^^,^^83^ have shown that incorporation multiple cell types with its complex cell-cell communication may hold the key to enhancing cardiac maturation and vascularization. One relevant key cell type that promotes the stabilization of vascular networks *in vivo* are the pericytes.^84^^,^^85^ With advancements in protocols to generate pericytes from iPSCs,^86^^,^^87^ their incorporation into tissue grafts may enhance vascular persistence and stability. Further studies are needed to demonstrate this *in vivo*. Finally, extending graft survival through bi-directional, graft-to-host and host-to-graft vascular anastomoses remains a critical challenge.

## Engineering perfusable vascularization in cardiac tissue models

Endothelial cellular networks in the heart form a seamless immunopermissive barrier and a vital lifeline to support trophic and tonic functions under physiological conditions. The paracrine capacity of ECs in secreting nitric oxide, growth factors, and cytokines regulates normal cardiac function. Similarly, the CM-EC crosstalk exerts a biomechanical influence as seen when the heart ECs’ protein expression changes dramatically with changes in cardiac loading, disrupting the mechanosensitive signaling and homeostasis.^88^^,^^89^ During an injury, pathological activation of endothelium facilitates immunosurveillance in the tissues, elevating the risk of inflammation-induced cardiovascular events.^90^ Hence endothelial cells are not only critical in the maintenance of vascular hemodynamics but also play a vital role in regulating the innate immune networks within the heart.^91^ Owing to the high metabolic demand of the heart, it requires a vasculature that can withstand different flow rates spanning multiple length scales. TE approaches have been employed in the fabrication of implantable arterial and small vessel tubular structures populated with hiPSC-vascular cells on synthetic and natural decellularized constructs. Synthetic vascular conduits generated using polyglycolic acid (PGA) fibers knitted into a tube format can withstand mechanical forces of cardiac motion and high pulsatile radial stress, and can be tuned to match the rupture pressure of veins (∼1500 mmHg) or arteries (∼3000 mmHg).^92^^,^^93^ For thick tissue transplantation, the generation of vascular grafts using allogenic cell sources may lead to immunogenic responses. An alternative strategy maybe to use less immunogenic pluripotent stem cell lines by suppressing human leukocyte antigen (HLA) class I and class II while over-expressing HLA-E gene.^94^ Capillaries composed of a single endothelial layer form highly dense vascular networks (>2000 capillaries mm^−3^) within the myocardium. Static co-culture systems can recapitulate endothelialization of 3D constructs with the spontaneous assembly of EC networks by aggregating defined cell-cell ratios.^95^^,^^96^ However, cardiac growth and angiogenesis *in vivo* is driven by intercellular interactions during development that promote tissue-specific features to the ECs. Compared to ECs from other organs, heart ECs exhibit a higher electrical resistance, metabolic rate, and angiogenic potential.^97^ Developmental organoid-like platforms achieved through co-differentiation of cardiomyocytes and non-myocytes offer CM-EC crosstalk reminiscent of that seen in myocardial morphogenesis.^40^^,^^98^

Efforts have also been made to perfuse the engineered heart tissues (EHTs) using sacrificial alginate fibers. In these constructs, murine cardiomyocytes, fibroblasts, smooth muscle cells, and endothelial cells self-assembled around the sacrificial template. The results suggested that a fine balance between flow rate and diffusion is critical for maintaining overall tissue quality.^99^ Despite development of protocols that allow endothelization of miniature cardiac tissues at an organoid scale, efforts to achieve higher vascularization density are limited by incomplete control over temporal growth factor concentrations and negative feedback from other cell populations.^100^ More importantly, the vascular networks formed within the self-assembled tissues are seldom assessed for perfusability and often regress within a short culture period. While there is no established consensus on the optimal duration to form “perfusable vascular networks” as opposed rudimentary endothelial branching, further studies are needed to optimize contributing factors, such as the presence of supportive peri-vascular cell types, growth factors, ECM, and culture conditions. Engineering hierarchical, perfusable vascular networks within multilayered architecture 3D bioprinting approaches has shown promise with enhanced control in placement of cells and ECM in all three dimensions and the generation of “ghost” channels using sacrificial bioinks to form embedded vascular networks. Using an extrusion-based technique, the sacrificial templating approach is shown to create perfusable, open-lumen structures in thick microtissues.^101^ Unlike microfluidic devices that are made of rigid polymers, the tunability of materials used in bioprinting allows fabrication of constructs capable of mimicking an abluminal ECM-like basement membrane and luminal flow-polarization. Most recently, a vascularized thick cardiac patch was created using 3D printing aided by computed tomography (CT) of a human heart to obtain anatomically precise dimensions. This printing methodology yielded perfusable, thick (∼2 mm) hydrogel patch comprising of iPSC-CMs and iPSC-ECs or neonatal cardiac cells with primary human ECs and fibroblasts. A sacrificial template was created by the simultaneous printing of omental hydrogel embedded with CMs and gelatin hydrogel embedded with endothelial cells, the liquefaction of which generated a ∼300 μm stable vascular tree with lumens within the functional, contracting cardiac patch.^102^

In a similar approach, using magnetic resonance imaging (MRI) of a human heart and extrusion-based bioprinting technique called freeform reversible embedding of suspended hydrogels (FRESH), multiscale vasculature was printed with collagen, showing large to smaller-scale vessels down to ∼100 μm in diameter.^103^ Another approach known as sacrificial writing into functional tissue (SWIFT) was used to print a bifurcating sacrificial channel in a slurry of cardiac spheroids composed of iPSC-CMs (∼80%) and primary fibroblasts (∼20%), followed by evacuation of the printed gelatin and perfusion of human umbilical vein endothelial cells (HUVECs) to form endothelial-lined channels with modest coverage. A luminal flow of 500 μL/min was achieved for the cardiac tissue, and after 8 days of perfusion evidence of sarcomeric remodeling was observed^104^ (the adult resting coronary blood flow is 800 μL/min/g of heart muscle^105^). Although these bioprinting methods utilize large number of myocardial cells and defined vasculature molding strategies, there is a clear lack of physiological maturity both in terms of overall tissue strain and vascular hierarchy comprising of capillaries, arterioles, and arteries. To achieve therapeutic benefit *in vivo*, it is not always essential to implant perfusable networks as they rapidly remodel in a highly metabolic *in vivo* environment. Cardiac patches fabricated using decellularized ECM and human c-kit+ and stomal cells patterned by a 3D bioprinting methodology has been shown to promote vasculogenesis in an immunocompromised rodent model, forming blood vessels with diameter of approximately 50 μm.^106^ The scale and form-factor of these engineered tissues may function *in vitro* or *in vivo* in small animal models. However, for human-scale tissues and translation it is key to develop protocols to obtain high fidelity, cardiac-specific ECs from iPSC sources that can form dense capillary networks with cardiac tissue-specific permeability.^107^ Such pioneering efforts are promising steps toward obtaining hierarchical microscale and macroscale vessels in a functioning cardiac patch that may be used for surgical anastomosis.

## Building functional innervation in cardiac models

The heart is highly vascularized and innervated by autonomic nerves. Innervation is key for modulating cardiac excitability. For example, sympathetic innervation promotes cell proliferation and physiological hypertrophy during development. Loss of sympathetic innervation dramatically stunts the growth of the heart.^108^^,^^109^ *In vivo*, neural crest cells (NCCs) develop from the neural plate and play an important role in the development of sympathetic and parasympathetic nervous systems of the heart. An orchestrated expression of bone morphogenetic proteins (BMPs) initiates the differentiation of NCCs that leads to autonomic neurogenesis, as well as maturation of cholinergic and noradrenergic precursors.^110^^,^^111^ Parasympathetic and sympathetic neurons share common precursors expressing mammalian achaete-scute homolog (MASH)-1.^112^ The MASH-1-induced expression of transcription factor paired-like homeobox (PHOX)-2A together with BMP-induced PHOX2B expression drive sympathetic neurons to release norepinephrine-synthesizing enzymes tyrosine hydroxylase (TH) and dopamine-β-hydroxylase (DBH).^113^ During early development, sympathetic, and parasympathetic neurons express shared markers and undergo distinct specification upon maturation.^114^^,^^115^ The divergence into parasympathetic cholinergic neurons is driven by downregulation of heart and neural crest derivatives that expressed 2 (*HAND2*) and GATA binding protein 3 (*GATA3*) to suppress a noradrenergic phenotype of the sympathetic ganglia.^114^ Once differentiated, these NCC-derived neurons are maintained by survival factors secreted by cardiomyocytes and non-myocytes.^116^^,^^117^ For example, the nerve growth factor (NGF) plays an important role in survival of both sympathetic and parasympathetic neurons and is more highly expressed in the densely innervated atrium than the ventricle.^118^^,^^119^ Neurotropin 3 (NT-3), another key neurotrophin expressed in cells adjacent to stellate ganglion (sympathetic neurons), stimulates axon guidance, the deletion of which results in shorter axonal projections from the stellate ganglion.^120^

Neighboring vascular and smooth muscle cells are also crucial in promoting the maturation of innervating neurons. For example, artemin is expressed by the endothelium and smooth muscle cells and acts on GFRα3, a glycosylphosphatidylinositol (GPI) receptor expressed by sympathetic neurons, to stimulate growth and integration with the cardiomyocytes.^121^ Other cell-secreted factors, such as endothelin-1 secreted by the venous endothelium,^122^ growth factors neuroligin expressed by FBs,^123^^,^^124^ and NGF by the vascular smooth muscle cells,^125^ mediate axonal guidance along the large vessels from the stellate ganglia to the myocardial layer. The innervating neurons, particularly the stellate ganglion neurons, undergo profound structural and electrophysiological remodeling in disease conditions, such as heart failure or arrhythmic events. Hence the incorporation of innervating cell types is essential for disease modeling and novel therapeutic targeting. Directed NCC differentiation from human ESCs and iPSCs using small molecules has allowed the generation of neural rosettes that express markers, such as SOX10 or p75/HNK1.^126^^,^^127^^,^^128^ As seen *in vivo* at the dorsal aorta, cytokine families such as BMPs, stromal cell-derived factor-1 (SDF1, a chemokine, also called CXCL12), and neuregulin1 (NRG1) further help in the specification from NCC to sympathetic ganglion neural cells expressing PHOX2B.^129^^,^^130^ Co-culturing of the sympathetic neurons with iPSC-derived cardiomyocytes led to better neurocardiac modulation in normal and pathological states.^131^^,^^132^

In parallel, studies show that *in vivo* cholinergic parasympathetic neurons originate from Schwann cell (SC) progenitors.^133^ Guided by this developmental pathway, directed differentiation approaches have been employed to obtain SC-derived parasympathetic neurons. NCC stimulated with NRG1 results in SC progenitors, which when cultured with a combination of neurotrophic factors (GDNF, BDNF, and CNTF) gives rise to functional parasympathetic neurite bundles.^134^^,^^135^^,^^136^ The resulting parasympathetic neurons when co-cultured with iPSC-CMs exhibited parasympathetic control upon cholinergic stimulation.^134^^,^^135^ Such model systems could be crucial in understanding neurocardiac physiology.^131^^,^^132^ For example, Long QT syndrome type 1 (LQT1) syndrome caused by loss-of-function mutation in the *KCNQ1* gene is essential in cardiac repolarization and is predominantly considered to be a myocyte defect. However, sympathetic neurons derived from human LQT1 iPSCs were shown to exhibit a higher firing propensity and frequency that correlated with the *KCNQ1* genotype.^131^ This suggests that sympathetic hyperactivity may play a supplementary role in triggering life-threatening arrhythmias in LQT1 that could lead to adverse cardiac events.

Similarly, iPSC-derived sympathetic neurons were also shown to mimic early pathology of diabetic neuropathy in high-glucose culture conditions that triggered hyperactivity and increased oxidative stress via accumulation of reactive oxygen species (ROS).^134^ Sympathetic neurons cocultured with human iPSC-derived CMs have been shown to enhance cardiac maturation by functional coupling,^129^ and the upregulation of genes associated with thin or thick myofiber filaments improved sarcomeric organization.^137^ Recently, using a bioengineering approach, Schneider et al. successfully incorporated innervated human cardiac muscle by fusing iPSC-derived sympathetic neural organoids and EHTs. After 6 weeks, neurofilaments from the autonomic neurons interlaced with the cardiomyocytes were found to form functional neuro-cardiac junctions.^138^ These iPSC-derived neurocardiac models incorporating sympathetic or parasympathetic cell types may prove critical to understanding cardiac pathophysiology relevant in congestive heart failure, sudden cardiac death, and polymorphic ventricular fibrillation.^139^

## Role of immune interactions in the heart: Balancing regeneration and remodeling

Tissue remodeling in cardiac disease or after injury is primarily activated by immune cells. For example, after cardiac injury, local inflammation recruits immune cells, such as polyfunctional monocytes and MΦs to phagocytose cellular debris.^140^^,^^141^ This enables fibroblast activation and migration, ECM deposition, and stabilization of the scar and ventricular function.^142^^,^^143^ In case of a sterile inflammation, the mobilization of the immune cells is triggered by release of damage associated molecular patterns (DAMPS) that regulate pro-inflammatory cytokines and chemokines in the heart.^144^ In an ischemic reperfusion injury, the cardiac mast cells are responsible for initiating an inflammation cascade that activates adjacent cells, such as the endothelium and MΦs. A further lack of blood supply will escalate the inflammation through introduction of activated complement proteins, neutrophil infiltration, and mast cell degranulation leading to cardiomyocyte cell death.^145^ Regulatory T cell activation and the secretion of TGF- β1, IL-10 (interleukin-10), and MMPs (matrix metalloproteinases) resolve the inflammatory damage by degrading ECM and limiting leukocyte infiltration.^146^ In later stages of ischemic injury, the MMP-cleaved active form of TGF-β1 binds to TGF type II receptor in fibroblasts to promote myofibroblast differentiation by secreting ECM proteins to maintain tissue integrity.^147^ Resident cardiac MΦs subsets that play an important role in tissue repair express major histocompatibility complex class II (MHC-II) and C-C chemokine receptor 2 (CCR2) to varying degrees.^148^^,^^149^ During aging and myocardial injury, CCR2+ MΦs replace the CCR2-subset.^149^ CCL2 is a chemokine essential for migration of CCR2+ MΦs, and the recruitment of CCR2+MHC-II^high^ MΦs assists in clearing cellular debris and presenting antigens.^150^^,^^151^

In addition to the resident cardiac MΦs, circulating monocytes expressing CCR2 differentiate into CCR2+ MΦs following migration into the myocardium. Beyond ischemic injury, models of cardiac inflammation in diseases like viral myocarditis provide valuable insights into immune cell dynamics in the heart. In these models, engineered platforms have been used to mimic hyperinflammatory environments caused by the infiltration of circulating monocytes. For example, several recent studies used a vascularized cardiac platform to model cardiac inflammation triggered by endothelial activation^47^ and the infiltration of circulating monocytes following SARS-CoV-2 infection.^77^ Animal studies have shown a preferential recruitment of Ly6c^high^ MΦs compared to the Ly6c^low^-expressing subset in the event of cardiac stress or injury.^152^^,^^153^ However, this distribution shifts in the later stages of injury, with an increase in Ly6C^low^ MΦs, which are typically non-inflammatory and involved in tissue repair.^153^^,^^154^ IL-1β (interleukin-1β), secreted by infiltrating MΦs during infarction or injury, exerts opposing effects on FBs. In the early stages, it suppresses the expression of α-smooth muscle actin (α-SMA) in FBs and delays their differentiation into myofibroblasts. In contrast, during later stages, IL-1β promotes the release of fibrotic mediators to drive ECM deposition. Single-cell RNA-seq analyses of the infarcted myocardium have revealed multiple subsets of highly plastic MΦs representing 64% of macrophage populations, which are phenotypically distinct from resident macrophage populations in steady state.^155^ The transition from the reparative to the regenerative phase following heart injury represents a balanced immune interplay between a fibrotic and a regenerative environment.

Macrophages derived from pluripotent sources are a valuable source to understand the immuno-cardiac crosstalk. Co-culturing LYVE1+ MΦs derived from human embryonic stem cells (hESCs) allowed them to integrate into engineered cardiac tissues with hESC-derived cardiomyocytes and primary FBs. The effective clearance ability of these primitive MΦs led to increased cardiomyocyte size, upregulation of sarcomeric proteins, and maturation of myosin isoforms mimicking the dynamics during early development.^156^ *In vivo* cardiac resident MΦs play a critical role in maintaining vascular stability and reducing cytotoxicity.^157^ This function was elegantly demonstrated using an engineered fibrin hydrogel embedded with iPSC-CMs, dental pulp stromal cells, HUVECs, and hESC-derived MΦs that was integrated into a perfusable iFlow platform.^158^ In the presence of MΦs, the system exhibited reduced permeability and a higher density of perfusable vessels over two weeks in culture compared to conditions without MΦs. Furthermore, the MΦs significantly upregulated pro-angiogenic cytokines and downregulated fibrosis-related genes, underscoring their homeostatic role in the absence of inflammation.^159^ iPSC-derived cardiac MΦs derived from hemogenic endothelium are more similar to tissue-resident-like MΦs. The incorporation of these MΦs in engineered cardiac tissues comprising of iPSC-CMs and iPSC-FBs has been shown to increase contractile force and β-adrenergic activity in iPSC-CMs.^83^ In another study, co-culturing iPSC-derived atrial cardiomyocytes with pro-inflammatory M1 MΦs revealed the contribution of inflammatory MΦs in triggering arrhythmias, including a strong correlation with atrial fibrillation patients based on gene expression.^160^ Such models are described in Table 2 and provide insights into the interactions between immune cells and other cardiac cell types in both healthy and diseased contexts.

**Table 2:** A summary 3D cardiac co-culture system with iPSC-derived neuronal cells and immune cells

Interface	Platform/Format	Tissue Dimensions	Basement membrane/Matrix	Cell numbers/Composition	Features/Response	Reference
Neuro-cardiac	iEHM (Fused loop-shaped Engineered Heart Muscle +Sympathetic Neuronal Organoids (SNOs)	∼4 mm × 1.5 mm	Collagen I/Matrigel	40% CMs, 32% FBs, 11% ECs, 6% pericytes, 3% neurons, 6% mesenchymal/progenitors and 1% monocytes/macrophages	⇑ Contractility, ⇑ sensitivity to nicotine, atropine, ⇓ chronotropy w/adrenergic blockade, capillaries (5–10 μm), arterioles (20–40 μm),	Schneider et al.^138^
Immuno-cardiac	EHT strip with microfluidic perfusion (InVADE)	∼1 mm × 0.6 mm	Fibrin, Gelatin	iPSC-CMs/FBs (50 × 10^6^ cells mL^−1^, HUVECs lined (25 × 10^6^ cells mL^−1^) (2:1), PBMCs (3 × 10^5^ cells mL^−1^)	Myocarditis model. ⇓ Contractility, ⇑ EC activation post viral infection triggered immune cell infiltration, and detection ⇑ circulating biomarkers	Lu et al.^77^
Immuno-cardiac	Biowire EHT strip	∼4 mm × 0.5 mm	Collagen I, Matrigel	hESC/hiPSC-CMs, hCFs, hESC-MΦs (75%:15%:10–20%)	MΦs promoted ⇑ tissue compaction, contractility, myocyte size, sarcomeric and desmosomal protein expression, ATP production, and ⇑ myocyte maturity	Hamidzada et al.^156^
Immuno-cardiac	Biowire EHT strip	∼4 mm × 0.5 mm	Fibrin	hESC-CMs, hESC-MΦs, HUVEC, Stromal cells (40:20:20:20)	MΦs promoted ⇑ contractility, active force, myocyte maturity, pro-angiogenic capacity of ECs and vessel stabilization via MMPs, ⇑ perfusion in cardiac tissues,	Landau et al.^159^
Immuno-cardiac	ECT strip	∼0.5 × 0.5 × 6 mm	Fibrin	hiPSC-CMs, hiPSC-FBs, hiPSC-MΦs (70%:23%:7%)	MΦs enhanced cardiac contractility, ⇑ myocyte size, force production, ⇑ β-adrenergic signaling	Lock et al.^83^

hESC, human embryonic stem cell; hiPSC, human induced pluripotent stem cell; EHT, engineered heart tissue; ECT, engineered cardiac tissue; iEHM, innervated engineered heart muscle; CM, cardiomyocyte; EC, endothelial cell; FB, fibroblast; HUVEC, human umbilical vein endothelial cell; hCF, human ventricular fibroblast; MΦs, macrophage; PBMC, peripheral blood mononuclear cell.

## From building blocks to the development of 3D chamber-like architecture using tissue engineering techniques

Many cardiac diseases are characterized by changes in cell morphology within the tissue architecture. Techniques such as microcontact printing allow the tuning of cardiomyocyte aspect ratio to mimic healthy versus diseases cell morphology. For example, in an infarcted myocardium with higher collagen deposition and crosslinking, there is a loss of myocyte alignment and remodeling of myocyte shape due to infiltrating scar tissue and activated fibroblasts.^161^ Geometric confinement with aspect ratios ranging from 1:1 to 7:1 has been shown to alter sarcomere alignment and morphology, such that increasing or decreasing the physiological aspect ratio of 7:1 leads to concentric or eccentric hypertrophy phenotype.^162^^,^^163^ Microcontact printing techniques have also provided insights into the effect of metabolic re-programming as a function of mechanical rigidity, cell shape, and fiber alignment by using a combination of hydrogel stamps functionalized with cell attachment peptides or ECM.^164^^,^^165^ Higher alignment generally augments functional readouts, such as calcium handling and conduction velocities compared to random orientations of the underlying substrate.

**Figure 3:**
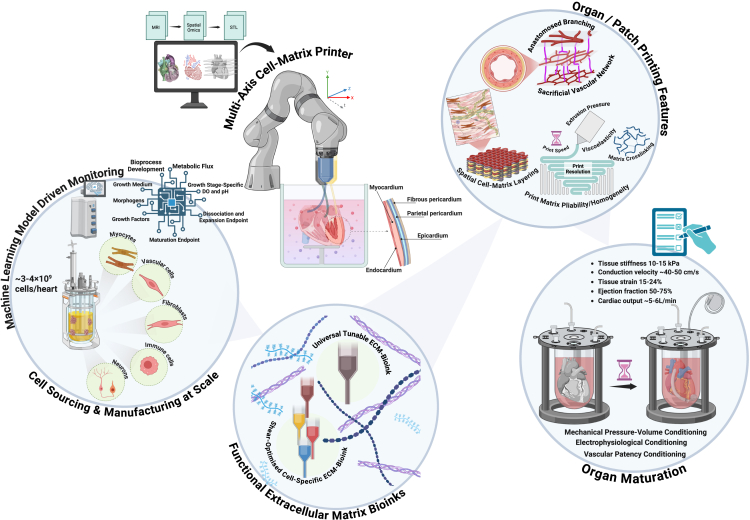
Roadmap for engineering a functional human heart via integrated biomanufacturing This schematic outlines the biofabrication pipeline for a human heart. First, iPSCs are differentiated into cardiomyocytes, vascular cells, fibroblasts, immune cells, and neuronal cells at clinical scale. These cells are embedded in extracellular matrix-based bioinks and assembled into an anatomically precise construct via multiaxial 3D bioprinting. The printed heart undergoes bioreactor conditioning with physiological pressure-volume cycles, electrical stimulation, and perfusion to achieve functional maturation in terms of stable vascular networks, native-like contractility, and tissue mechanics.

To engineer human cardiac tissue, a comprehensive understanding of its structural and functional hierarchy, particularly how specialized cellular populations and their three-dimensional organization enable coordinated contractility, is necessary. The heart’s four-chambered architecture comprises atrial and ventricular compartments separated by valvular structures that enforce unidirectional hemodynamic flow. While protocols exist for differentiating certain cardiac lineages from iPSCs at large-scale,^16^ bioprinting of high-purity populations of specialized conduction cells (e.g., sinoatrial nodal or Purkinje cells) for sequential cardiomyocyte contraction via precisely patterned cellular tracks has not been achieved. Achieving such a task requires: (1) high-throughput bioprinters capable of rapid, large-scale deposition without compromising cell viability; (2) cell-specific bioinks optimized for cardiomyocyte alignment, endothelial network formation, and conductive fibroblast integration; (3) electromechanical coupling of spatially orchestrated conduction system exhibiting adult-like electrophysiology; (4) stimuli-responsive biomaterials that enable tissue compaction and vascular anastomosis post-printing; and (5) advanced bioreactor systems for electromechanical conditioning to achieve physiological maturation (Figure 3). Combining these approaches to engineer adult-like cardiac constructs will serve as a framework to set clinical standards for human transplantation. The development of novel hybrid materials and cytocompatible bioinks has advanced bioprinting approaches for cardiac tissue engineering. These approaches generally involve the creation of a blueprint of the tissue architecture obtained from medical imaging data, followed by the use of high viscosity hydrogel to achieve high-resolution printing performance while incorporating mechanical properties of the tissue and viability of the cells. An engineered healthy myocardium tissue should possess anisotropy with up to 25% bi-axial deformation and stiffness ranging from 12 to 20 kPa.^166^ Hydrogel materials popularly used for bioprinting approaches are made of hydrophilic polymers derived from natural sources or are artificially synthesized, which are then crosslinked to form mechanically pliable, porous structures. The versatility of the synthetic or semi-synthetic polymers also provides several advantages in tuning mechanical properties, particularly in fabricating complex volumetric structures (Table 3). One such material that has been extensively used in bioprinting is gelatin methacrylate (GelMA), which is known for its cellular compatibility and programmable physical and chemical features. Using micro-continuous optical printing technology, several studies have demonstrated encapsulation of cardiomyocytes in GelMA hydrogel lines between printed pillars to generate microtissues.^167^^,^^168^ The printing resolution for such modalities can be adjusted to achieve higher positioning densities to form continuous anisotropic, interlaced tissues, with distances between the adjacent strips being limited to 30 μm or less to support higher electrical propagation and synchronized contractility.^169^

**Table 3:** A summary of biofabrication methodologies to generate volumetric, multi-layered cardiac tissue assemblies using human pluripotent stem cell-derived cardiac cells

Model	Printing Method	Cell seeding	Structure/Function/Cell numbers					Cell composition	Support Matrix	Reference
			Format	Size	Stiffness/Strain	Pressure/Output volume	Cell numbers			
Ventricle	Balloon catheter molding	Post-fabrication	Single chamber	∼6 mm (W), ∼5 mm (H)	–	∼0.09 mmHg/∼4.8μL	∼0.9 × 10^7^ CMs ml^−1^, ∼1 × 10^6^ FBs ml^−1^	hiPSC-CMs,HDFs	Agarose/Collagen/Matrigel	Li et al.^170^
Ventricle	Rotary jet electrospinning	Post-fabrication	Single/Dual chamber	∼5.5 mm (ID), ∼8 mm (H)	∼325 kPa/0.04–0.12%	∼0.006 mmHg/∼0.1μL	1–1.4 × 10^7^/single chamber, 2.5 × 10^7^/dual chamber	hiPSC-CMs	PCL/Gelatin	Chang et al.^171^
Ventricle	Pull spinning	Post-fabrication	Single chamber	∼9 mm (ID), ∼10 mm (H),	∼500 kPa	∼0.04 mmHg/∼480 μL	∼2.3 × 10^6^/construct	hiPSC-CMs	PCL/Gelatin	MacQueen et al.^172^
Ventricle	Extrusion printing without support bath	Post-fabrication	Single chamber	∼10 mm (W), ∼10 mm (H)	∼51 kPa	–	∼8 × 10^6^/construct	hiPSC-CMs	Alginate/Gelatin	Choi et al.^173^
Ventricle	Extrusion-printing with support bath/FRESH	Co-printed	Single chamber	∼6 mm (ID), ∼8 mm (H)	–	–	>300 × 10^6^ mL^−1^	hESC-CMs, hCFs (49:1)	Collagen I/Fibrin	Lee et al.^103^
Ventricle	Extrusion-printing with support bath/FRESH	Co-printed	Dual chamber	∼12 mm (W), ∼15 mm (H)	∼6 kPa	0.23 mmHg/0.6μL	∼15 × 10^6^ mL^−1^	hiPSC-CMs	Collagen methacrylate, Gelatin methacrylate, Fibronectin, Laminin-III	Kupfer et al.^174^
Ventricle	Extrusion-printing with support	Co-printed	Dual chamber	∼14 mm (D), 20 mm (H)	∼900 Pa	–	1 × 10^8^ mL^−1^ CMs, 3 × 10^6^ mL^−1^ ECs	hiPSC-CMs, hiPSC-ECs	Decellularized omenta matrix	Noor et al.^102^
Perfusion Chamber mold	Extrusion-printing with support bath/SWIFT	Co-printed	Silicone mold/Perfusion chamber	6 mm (Top W), 4.2 mm (Bottom W), 4.2 mm (D), 12 mm (H)	∼200 Pa	–	∼2.4 × 10^8^ mL^−1^ CMs, 6 × 10^5^ mL^−1^ HDFs	hiPSC-CMs, neonatal HDF	Collagen I, Matrigel, Gelatin	Skylar-Scott et al.^104^

hESC, human embryonic stem cell; hiPSC, human induced pluripotent stem cell; CM, cardiomyocyte; EC, endothelial cell; FB, fibroblast; HDF, human dermal fibroblast; hCFs, human ventricular fibroblast; HUVEC, human umbilical vein endothelial cell.

Bioprinting approaches have generated scalable arrays using scaffold-free patterning and modular assembly of anisotropic building blocks with gelatin and fibrinogen hydrogels, which could revolutionize high-throughput *in-situ* drug screening.^63^^,^^175^^,^^176^ One of the major requirements to build a whole human heart is the fabrication of a vascularized myocardial chambers (∼4–10 mm thick) that can accurately replicates anatomical and cellular composition capable of sustaining 17%–28% strain, high conduction velocity (∼50 cm/s), and a cardiac output of approximately 5–6 L/min.^177^^,^^178^ Advancements in fiber manufacturing technologies such as melt electrospinning, solution electrospinning, rotary jet electrospinning, and pull spinning have overcome this limitation by controlling anisotropy and volumetric design features. These new technologies allow “weaving” of the fibers into 3D structures that resemble the heart’s geometry and features. For example, researchers have successfully fabricated a ventricle-like fibrous scaffold using a gelatin-polymer composite with a chamber volume of ∼500 μL.^179^ The ventricle with a tensile strength of ∼500 kPa was able to support integration of human iPSC-CMs and rat myocytes, though the study also found poor chronotropy, ejection fraction, and contractile work due to low cell density and an immature phenotype. In an iterative follow-up study, Chang et al. utilized the rotary jet electrospinning technique to form 3D ellipsoid-shaped single- and dual-chamber models with circumferential and helical patterns using polycaprolactone and gelatin fibers.^171^ Both single and dual ventricle chambers were seeded with hiPSC-CMs for conduction and deformation measurements. Overall, the construct was still limited in obtaining native matrix stiffness and strain by several orders of magnitude. However, the cell-seeded ventricle that was helically spun showed a promising longitudinal conduction velocity of ∼19 cm/s, providing further evidence that helically aligned tissue is correlated with optimal cardiac work.

Despite the success in obtaining high degree of anisotropy and stiffness using electrospinning and 3D-printed free-standing structures,^173^ one disadvantage of scaffold pre-fabrication using synthetic matrices crosslinked prior to cell seeding is that it may impede progressive stretch yielding tissues, which is needed for structural and functional myocyte maturation over time. Extrusion-based printing helps overcome the cell seeding challenge by simultaneous extrusion of cell-matrix “bioinks” via an extrusion nozzle, allowing optimal embedment under controlled pressure and shear stress. This technique allows printing of complex and high-resolution tissue structures in a sacrificial support matrix, which can be thermally or enzymatically manipulated after the tissue, achieves structural stability. Using an extrusion-based bioprinting technique called FRESH, Lee et al. fabricated a left ventricle model by forming an ellipsoidal shell (∼6 mm inner diameter, 8 mm in height) with the outer and inner layers made of collagen, with hESC-CMs and 2% primary human FBs sandwiched in a collagen-based bioink between the two layers. After seven days, the ventricles showed spontaneous contraction with anisotropic calcium wave propagation and a conduction velocity of ∼2 cm/s along the long axis upon point stimulation.^103^ Employing the FRESH methodology, Kupfer et al. fabricated a dual chamber model with a one-way flow loop; one of the unique features of this model was the use of multiple ECM proteins to facilitate cell-driven remodeling.^174^ The chamber seeded with iPSC-CMs was maintained for 6 weeks and could generate a chamber pressure of 0.2 mmHg, higher than values reported to date in a similar model.

In the myocardium, mechanical coupling between cells and the compliant matrix contributes to physiological hypertrophy. During development, compliance changes in the ECM results in stretch-mediated local stresses and strains around the myocytes.^180^ The cumulative resistance generated from tugging at the ECM by coupled, contracting myocytes establishes the tension that defines the force of contraction and subsequently cardiac output.^181^ Employing programmable matrix that can co-mature along with the myocytes and non-myocytes may therefore significantly enhance the performance of bioengineered constructs. In a proof-of-concept study, a fully personalized, small-scale heart (14 mm in diameter and 20 mm in height) was fabricated using 3D bioprinting. This rat-sized heart was composed of human iPSC-CMs and iPSC-ECs, embedded in a customized bioink formulation derived from the patient’s fatty tissue to enhance immunocompatibility.^102^ From a manufacturing perspective, one challenge in the generation of thick, functional tissue is to manufacture billions of cells in a cost-effective manner. Several recent efforts in large-scale production and cryopreservation of cardiomyocytes have shown promise with novel techniques that may yield billions of cells for bioprinting on full-scale heart scaffolds.^16^^,^^182^ These new approaches may help us further understand the cardiac structure-function relationships, but challenges remain, including the need to incorporate programmable materials that can remodel with strain adaptation, vascularization, innervation, and overall tissue compaction.

## Challenges and conclusion

The journey toward creating functional, transplantable cardiac tissues remains an ambitious goal in the field of tissue engineering. While significant strides have been made, key challenges persist, particularly in replicating hierarchical vasculature, functional innervation, and innate immune integration. These elements are critical not only for the physiological performance of engineered tissues but also for their long-term viability and adaptability *in vivo*.

The vascularization challenge has long been recognized as a critical bottleneck. Although progress in microfluidic technology and biofabrication methods, including 3D bioprinting and sacrificial templating, has facilitated the creation of perfusable structures, these approaches remain predominantly restricted to small-scale applications.^9^ The creation of larger, human-scale tissues requires the ability to mimic capillary networks and higher-order vascular structures to support adequate perfusion and metabolic demands. Solving this challenge demands a deeper understanding of vascular biology and the further development of scalable fabrication technologies.

Incorporating functional innervation and immune interactions into engineered tissues is equally vital. Neural integration is essential for the modulation of cardiac rhythm and response to physiological stressors, and immune components also play key roles in tissue homeostasis, repair, and adaptation. Progress in iPSC-based differentiation protocols has enabled the generation of cell types that recapitulate aspects of neurocardiac and immuno-cardiac interactions, but these remain only early steps toward fully integrated systems at the present. Addressing these challenges will require a multidisciplinary, collaborative approach that spans the fields of biology, engineering, and clinical practice. Innovations in biomaterials, bioprinting, and computational modeling, coupled with emerging insights into cellular and molecular mechanisms, offer promising pathways forward. The recent recognition of organ-on-chip systems as a viable alternative to animal models by regulatory bodies like the FDA underscores the transformative potential of these approaches in drug development and disease modeling efforts.^3^^,^^183^ Moreover, these organ-on chip models provide crucial insights into the mechanisms of vascular and immune development in both healthy and diseased states.

Finally, a concerted, “moonshot-style” effort to address these challenges could further accelerate the development of transplant-grade implantable cardiac tissues or even a fully functional organ. Achieving this goal will require sustained research investment and cross-disciplinary collaboration to manufacture personalized, clinical-grade cardiac tissue replacements capable of revolutionizing cardiovascular medicine, and improve patient outcomes.

## Acknowledgments

We thank Blake Wu for critical reading of the article and feedback. Manuscript figures were created with BioRender.com. This project was supported by grants from the 10.13039/100000002National Institutes of Health R01 HL141851, R01 HL150693, R01 HL163680, UG3 TR005845, and U01 AI183953 (J.C.W.); K99 HL163443 (D.T.); Department of Energy DE-SC0025363 (J.C.W.); and 10.13039/100000104National Aeronautics and Space Administration 80ARC022CA003 (J.C.W.).

## Author contributions

D.T. and J.C.W. conceptualized, wrote, reviewed, and revised the manuscript.

## Declaration of interests

J.C.W. is co-founder and scientific advisory board member of Greenstone Biosciences.
